# MicroRNA-663a is downregulated in non-small cell lung cancer and inhibits proliferation and invasion by targeting JunD

**DOI:** 10.1186/s12885-016-2350-x

**Published:** 2016-05-16

**Authors:** Yi Zhang, Xiaoman Xu, Meng Zhang, Xin Wang, Xue Bai, Hui Li, Liang Kan, Yong Zhou, Huiyan Niu, Ping He

**Affiliations:** Department of Geriatrics, Shengjing Hospital of China Medical University, 36 Sanhao Road, Shenyang, 110004 China; Department of Respiratory Medicine, Shengjing Hospital of China Medical University, Shenyang, China

**Keywords:** Lung cancer, miR-663a, JunD, Proliferation, Invasion

## Abstract

**Background:**

MicroRNA-663a expression is downregulated in several tumors. However, its functions and mechanisms in human non-small cell lung (NSCLC) cancer remain obscure. The present study aimed to identify the expression pattern, biological roles and potential mechanisms by which miR-663a dysregulation is associated with NSCLC.

**Methods:**

We examined expression level of miR-663a in 62 cases of NSCLC tissues and 5 NSCLC cell lines by reverse transcription PCR. In vitro, gain-of-function and loss-of-function experiments were performed to examine the impact of miR-663a on proliferation, cell cycle progression and invasion of NSCLC cells. Using fluorescence reporter assays, we also explored the potential targets and possible mechanisms of miR-663a in NSCLC cells.

**Results:**

Downregulation of miR-663a was observed in 42 of 62 of lung cancer tissues compared with paired normal tissues (mean cancer/normal value = 0.745) and its downregulation correlated with nodal metastasis. Transfection of miR-663a mimic suppressed cell proliferation, cell cycle progression and invasion, with downregulation of cyclin D1, cyclin E and MMP9 in both H460 and H1299 cell lines. Transfection of miR-663a inhibitor in both H460 and H1299 cell lines exhibited the opposite effects. In addition, we confirmed that miR-663a could inhibit AP-1 activity and AP-1 component JunD was a direct target of miR-663a in lung cancer cells. Transfection of miR-663a mimic downregulated JunD expression. In addition, JunD siRNA treatment abrogated miR-663a inhibitor-induced expression of cyclin D1, cyclin E and MMP9. Above all, both miRNA mimic and inhibitor in two different NSCLC cell lines demonstrated that miR-663a inhibits proliferation and invasion by targeting AP-1 transcription factor JunD.

**Conclusions:**

This study indicates that miR-663a downregulation might be associated with NSCLC progression. MiR-663a suppresses proliferation and invasion by targeting AP-1 component JunD in NSCLC cells.

**Electronic supplementary material:**

The online version of this article (doi:10.1186/s12885-016-2350-x) contains supplementary material, which is available to authorized users.

## Background

Lung cancer is the leading cause of cancer-related death worldwide, and the incidence of lung cancer is increasing [1]. Overall, the 5-year survival rate has remained at 15 % for the past two decades. Although targeted therapies have been established, genetic mutations causing activation of these gene products are identified only in a limited number of cancers [2, 3]. On the other hand, a variety of complex genetic, epigenetic, and microenvironmental factors play important roles in survival and invasion of tumor cells [4–7]. Hence, identification of these biological factors and elucidation of their regulatory pathways in governing tumor development, invasion, and metastasis is an important step toward the rational design of drugs for treatment of advanced NSCLC.

MicroRNAs (miRNAs) are a class of small non-coding RNAs, approximately 20–25 nucleotides, which regulate gene expression post-transcriptionally. Nearly 50 % of human miRNAs are located at fragile sites and genomic regions involved in cancers [8–10]. Emerging evidence shows that miRNA dysregulation is associated with various cancers including lung cancer [10, 11]. Previous studies have shown that miR-663a, a member of primate-specific miRNA family, is associated with a variety of important biologic processes such as viral infection, inflammatory responses and autoimmune diseases [12, 13]. However, its role in tumor progression is controversial. miR-663a serves as a potential tumor suppressor in gastric cancer, colorectal carcinomaand acute lymphoblastic leukemia [14–17], while it acts as an oncogene in nasopharyngeal carcinoma and breast cancer [18, 19].

In the present study, we evaluated miR-663a expression and clinical relevance in human non-small cell lung cancer tissues. Its involvement in biological behavior and the underlying molecular mechanisms were also investigated. Our data identified miR-663a as a potential tumor suppressor in human lung non-small cell lung cancer.

## Methods

### Samples

Fresh samples from lung cancer and corresponding normal adjacent tissue were obtained from patients at Shengjing of China Medical University between January 2011 and November 2013 with informed consent. None of the patients in the study received any chemotherapy or radiation therapy before surgery. This study was conducted with the approval of the Ethics Committee at Shengjing Hospital of China Medical University. Written informed consent was obtained from all patients. Research carried out is in compliance with the Helsinki Declaration. Tumor samples were stored at -80 °C for RNA extraction (the percentage of tumor tissue was >90 %).

### Cell culture, reagents and transfection

HBE135, H1299, H157, H1395, H460 and H3255 cell lines were obtained from American Type Culture Collection (Manassas, VA, USA). Cells were cultured in RPMI-1640 (Invitrogen, Carlsbad, CA, USA) containing 10 % fetal calf serum (Invitrogen, Carlsbad, CA, USA), 100 IU/ml penicillin (Sigma, St. Louis, MO, USA), and 100 μg/ml streptomycin (Sigma). Cells were grown on sterilized culture dishes and were passaged every 2 days with 0.25 % trypsin (Invitrogen, Carlsbad, CA, USA).

A mimic negative control, miR-663a mimic, an inhibitor negative control and a miR-663a inhibitor were purchased from RiboBio (Guangzhou, China). miRNA mimic is small, double-stranded RNA molecule, designed to mimic endogenous mature miRNA molecules when transfected into cells. miRNA inhibitor is small, single-stranded RNA with chemical modification that regulate gene expression by binding to and inhibiting a specific mature miRNA. MiR-663a mimic and inhibitor were transfected using Dharmafect1 Transfection Reagent (Dharmacon Lafayette, CO, USA). Briefly, complexes containing the mimic or inhibitor were prepared according to the manufacturer’s protocol and then cells were transfected with mimic negative control (100nM), miR-663a mimic (100nM), inhibitor control (200nM) or miR-663a inhibitor (200nM), respectively.

siGENOME SMARTpool siRNA for JunD and Non-targeting siRNA #1 were purchased from Dharmacon (100nM per well). The cells were transfected with siRNA using the DharmaFECT 1 (0.20 μL/well; Dharmacon Lafayette, CO, USA) according to the manufacturer’s protocol.

### Quantitative real-time PCR

Total RNA was extracted from fresh tissues and cells using Trizol (Invitrogen, Carlsbad, CA, USA) according to the manufacturer’s instructions. Quantitative real-time PCR was performed using SYBR Green PCR master mix (Applied Biosystems, Foster City, CA, USA) on 7900HT Fast Real-Time PCR System (Applied Biosystems, Foster City, CA, USA). Primers for miR-663a (Bulge-LoopTM miRNA qRT-PCR Primer Set for has-miR-663a, RiboBio, Guangzhou, China) and U6 (U6 snRNA qRT-PCR Primer Set, RiboBio, Guangzhou, China) were used for PCR analysis in accordance with the manufacturer’s protocol. Other primers are as followers: cyclin D1 forward, 5′-GCTGGAGGTCTGCGAGGA-3′, cyclin D1 reverse, 5′-ACAGGAAGCGGTCCAGGTAGT-3, cyclin E forward, 5′-AGCCAGCCTTGGGACAATAAT-3′, cyclin E reverse, 5′-GAGCCTCTGGATGGTGCAAT-3′, p21 forward, 5′-CCTCATCCCGTGTTCTCCTTT-3′. p21 reverse, 5′-GTACCACCCAGCGGACAAGT-3′. CDK4 forward, 5′-CCGAAGTTCTTCTGCAGTCC-3′. CDK4 reverse, 5′-GTCGGCTTCAGAGTTTCCAC-3′. CDK6 forward, 5′-GTGACCAGCAGCGGACAAAT-3′. CDK6 reverse, 5′-CCACAGCGTGACGACCACT-3′. MMP2 forward, 5′-TGTGTTCTTTGCAGGGAATGAAT-3′. MMP2 reverse, 5′-TGTCTTCTTGTTTTTGCTCCAGTTA-3′. MMP9 forward, 5′-CCTCTGGAGGTTCGACGTGA-3′, MMP9 reverse, 5′-TAGGCTTTCTCTCGGTACTGGAA-3′, β-actin forward, 5′-ATAGCACAGCCTGGATAGCAACGTAC-3′, β-actin reverse, 5′-CACCTTCTACAATGAGCTGCGTGTG-3′. A dissociation step was performed to generate a melting curve to confirm the specificity of the amplification. Each PCR analysis was performed in triplicate. The relative levels of gene expression were represented as ΔCt = Ct gene –Ct reference, and the fold change of gene expression was calculated by the 2^-ΔΔCt^ method.

### Western blot analysis

Total proteins from cells were extracted in lysis buffer (Pierce, Rockford, IL, USA) and quantified using the Bradford method. 50 μg protein was separated by SDS-PAGE. Samples were transferred to polyvinylidene fluoride membranes (Millipore, Billerica, MA, USA) and incubated overnight at 4 °C with antibody against cyclin D1(1:1000, Cell Signaling Technology, Boston, MA, USA), cyclin E (1:1000, Cell Signaling Technology, Boston, MA, USA), p21(1:1000, Cell Signaling Technology, Boston, MA, USA), CDK4(1:1000, Cell Signaling Technology, Boston, MA, USA), CDK6 (1:1000, Cell Signaling Technology, Boston, MA, USA), MMP2(1:1000, Cell Signaling Technology, Boston, MA, USA), MMP9(1:1000, Cell Signaling Technology, Boston, MA, USA) and JunD (1:1000, Cell Signaling Technology, Boston, MA, USA) and GAPDH (1:1000; Santa Cruz, CA, USA). After incubation with peroxidase-coupled anti-mouse/rabbit IgG (1:1000, Cell Signaling Technology, Boston, MA, USA) at 37 °C for 2 h, bound proteins were visualized using ECL (Pierce, Rockford, IL, USA) and detected using a DNR BioImaging System (DNR, Jerusalem, Israel). Relative protein levels were quantified using ImageJ software.

### Colony formation assay

For evaluation of colony formation, cells were transfected for 48 h before being seeded into 6-cm cell culture dishes (800 per dish) and incubated for 14 days. The plates were washed with PBS and the colonies were stained with the Giemsa dye. The number of colonies with more than 50 cells was manually counted under the microscope.

### Cell cycle analysis by flow cytometry

Cells (500,000) were seeded into 6 cm tissue culture dishes and cultured overnight. Forty eight hours after transfection, cells were harvested, fixed in cold 70 % ethanol washed with phosphate-buffered saline (PBS) and stained with 5 mg/ml propidium iodide in PBS supplemented with RNase A (Roche, Indianapolis, IN) for 30 min at room temperature. The cells were analyzed by flow cytometer (Becton-Dickinson and Co., San Jose, CA, USA). One-parameter histogram was plotted according to the distribution of nuclear DNA content in each cell detected by flow cytometer. Cells in each individual phase of the cell cycle were determined based on their DNA ploidy profile.

### Matrigel invasion assay

Cell invasion assay was performed using 24-well transwell chambers with pore size of 8 μm, and the inserts were coated with 20 μl Matrigel (1:3 dilution, BD Bioscience, San Jose, CA, USA). Sixty hours after transfection, cells were trypsinized, transferred to the upper matrigel chamber in 100 μl serum-free medium containing 3 × 105 cells, and incubated for 18 h. Medium supplemented with 10 % FBS was added to the lower chamber as the chemoattractant. Then, the non-invading cells on the upper membrane surface were removed with a cotton tip, and the cells that passed through the filter were fixed in 4 % paraformaldehyde and stained with hematoxylin. The experiments were performed in triplicate.

### Luciferase reporter assay

Reporter gene transfection and luciferase activity assay were performed as follows: cells in confluent growth on a 24 well plate were co-transfected with the firefly luciferase reporter (0.2 μg) along with the Renilla luciferase reporter (0.02 μg), which was used for normalization, using Attractene reagent (Qiagen, Hilden, Germany) according to the protocols provided by the manufacturers. The reporter plasmids of AP-1 were purchased from Biotime Biotechnology, China. The pAP1-Luc contains SV40 early enhancer/promoter. The AP-1 response element was listed as follows: GGCCTAACTG GCCGGTACCG CTAGC**TGACT AATGACTAAT GACTAATGAC;** CCGGATTGAC CGGCCATGGC GATCG**ACTGA TTACTGATTA CTGATTACTG**. The luciferase activity was measured in cellular extracts using a dual luciferase reported gene assay kit (Promega, San Luis Obispo, CA, USA). The relative activity of the reporter gene was calculated by dividing the signals from firefly luciferase reporter by the signals obtained from Renilla luciferase reporter.

### Validation for interaction of miRNAs and Target Genes using Luciferase Reporter Assays

A pmiR-RB-REPORT™ vector (Ribobio Co., Guangzhou, China) was used for 3′ UTR-luciferase reporter assays to detect interactions of miR-663a with JunD (Gene ID: 3727). The TargetScan Human database 6.2 http://targetscan.org/was used to identify miRNA binding sites. The Wild-type miR-663a target site in JunD 3′-untranslated region was CCCCGCC. The mutant miR-663a target site was CGGGCGC. The miR-663a mimic, miR-663a inhibitor and their corresponding negative controls (Ribobio Co. Guangzhou, China) were transfected with pmiR-RB-REPORT™-target gene UTR. Reporter assays were conducted in triplicate. The cells were co-transfected with 100 nM mimic and 100 ng/mL reporter plasmid by attractene agent (Qiagen, Hilden, Germany) according to the manufacturer’s instructions

### Statistical analysis

SPSS version 11.5 for Windows was used for all statistical analyses. Student’s *t*-test was used to compare densitometry data on focus numbers between control and treated cells. All p values are based on a two-sided statistical analysis and *p* < 0.05 was considered to indicate statistical significance.

## Results

### miR-663a expression was downregulated in NSCLC and associated with lymph lode metastasis

**Fig. 1:**
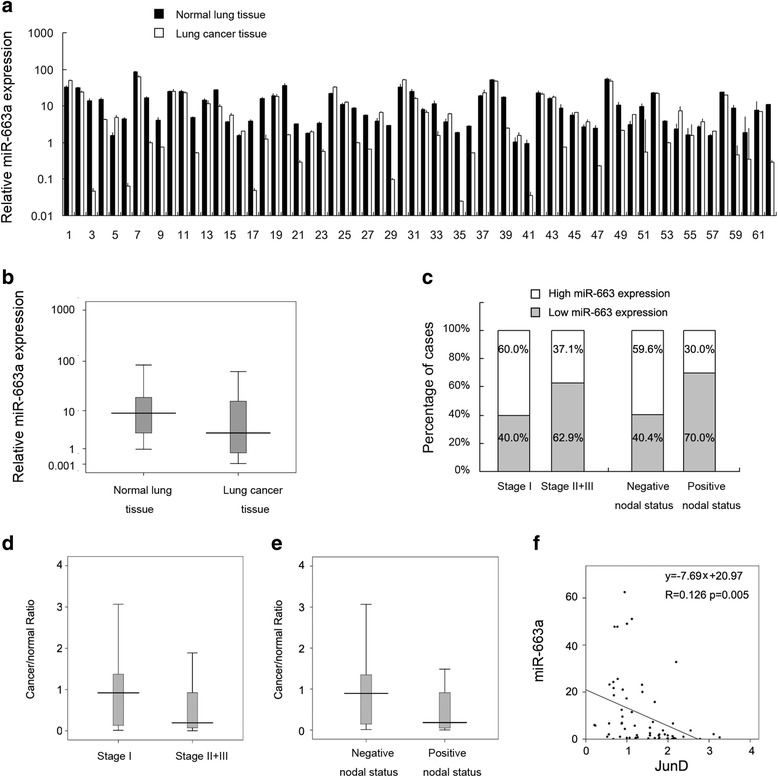
Low expression level of miR-663a in lung cancer patients. **a** Paired bar chart showing relative expression of 62 fresh NSCLC tissues and paired normal tissues. **b** Boxplot indicates mean miR-663a levels in NSCLC tissues and paired normal tissues. **c** Bar chart showing correlation between miR-663a expression level and TNM stage, lymph node status of 62 fresh paired samples. In stageI NSCLC, the percentage of high miR-663a expression was 60 % and in stageII-III NSCLC, the percentage was 37.1 %. In NSCLC without nodal metastasis, the percentage of high miR-663a was 59.6 % and in NSCLC without nodal metastasis, the percentage was 30 %. **d** Boxplot indicates mean cancer/normal miR-663a ratio in stageI and stageII-III NSCLC tissues. **e** Boxplot indicates mean cancer/normal miR-663a ratio in NSCLC tissues with or without nodal metastasis. **f** Relationship between miR-663a and JunD mRNA expression in NSCLC tissues (*p* = 0.005)

We examined levels of miR-663a by RT-qPCR in 62 fresh NSCLC tissue samples with paired normal lung tissues. As shown in Fig. 1a, the expression levels of miR-663a were downregulated in 42 of 62 NSCLC tissues. As shown in the boxplot (Fig. 1b), the mean level of miR-663a was lower in NSCLC tissues than in normal lung tissues (mean cancer/normal value = 0.745). To determine the effect of miR-663a on tumor progression, NSCLC patients were divided into two groups, low and high expression, according to the median relative expression level of miR-663a in cancerous tissues (median cancer/normal value = 0.773).

Correlations between miR-663a expression and clinicopathologic variables were shown in Table 1. Significant correlation was observed between miR-663a expression and nodal status (*p* = 0.0297) (Chi-Square test). In 20 cases presenting with positive nodal status, 14 (70.0 %) cases had low expression of miR-663a, while the low expression rate was 17/42 (40.4 %) in tumor samples without nodal metastasis (Fig. 1c-e). No correlation was observed between miR-663a expression and gender (*p* = 0.7668), age (*p* = 0.7926), histology type (*p* = 0.7978), stage (*p* = 0.0730) or histological grade (*p* = 0.3508).

**Table 1:** Distribution of miR-663a status in NSCLC according to clinicopathological characteristics

Characteristics	Number of patients	Low miR-663a expression	High miR-663a expression	*P*
Age				
< 60	23	12	11	0.7926
≥ 60	39	19	20	
Gender				
Male	47	24	23	0.7668
Female	15	7	8	
Histology				
Adenocarcinoma	27	13	14	0.7978
Squamous cell carcinoma	35	18	17	
Differentiation				
Well	10	7	3	0.3508
Moderate	42	20	22	
Poor	10	4	6	
TNM stage				
I	35	14	21	0.0730
II + III	27	17	10	
Tumor status				
T1	16	7	9	0.5616
T2 - T4	46	24	22	
Nodal status				
N0	42	17	25	0.0297
N1 - N3	20	14	6	

### miR-663a regulates cell proliferation and invasion in vitro

**Fig. 2:**
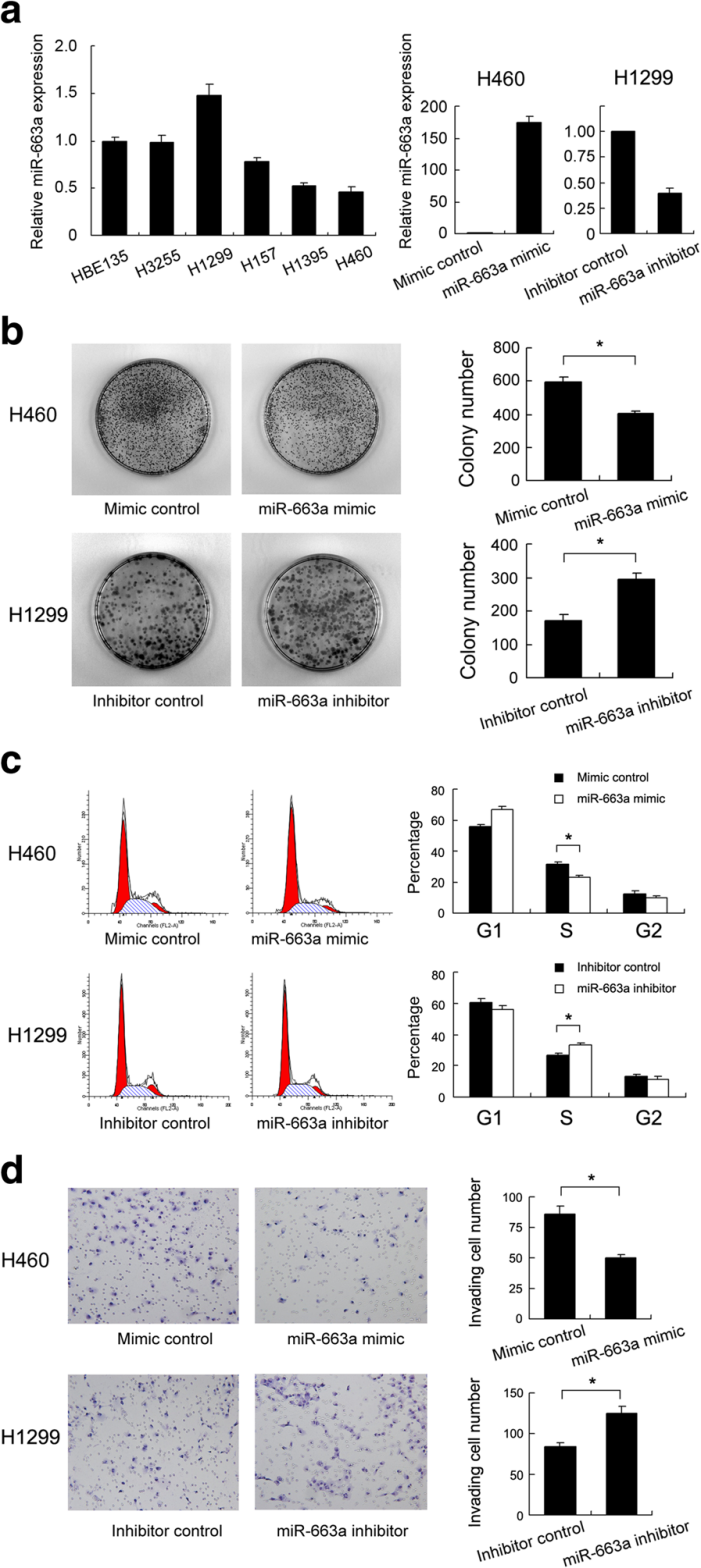
Effects of miR-663a on proliferation and invasion in NSCLC cells. **a** Expression of miR-663a in 5 NSCLC cell lines (H1299, H3255, H460, H1395, H157) and normal bronchial cell line HBE135. miR-663a mimic significantly upregulated miR-663a level in H460 cells and its inhibitor downregulated miR-663a expression in H1299 cells. **b** Colony number of H460 cells transfected with miR-663a mimic was lower than cells transfected with control (control 596 ± 28 vs mimic 401 ± 18, *p* < 0.001). Colony number of H1299 transfected with miR-663a inhibitor was higher than cells transfected with control (control 171 ± 16 vs inhibitor 295 ± 18, *p* < 0.001). **c** miR-663a mimic increased G1 phase percentage and decreased S phase percentage in H460 cells. miR-663a inhibitor decreased G1 phase percentage and increased S phase percentage in H1299 cells. **d** Matrigel invasion assay showed that invading number of H460 transfected with miR-663a mimic was lower than cells transfected with control (control 86 ± 6 vs mimic 50 ± 3, *p* < 0.001). Invading number of H1299 cells transfected with miR-663a inhibitor was higher than cells transfected with control (control 84 ± 5 vs inhibitor 125 ± 9, *p* < 0.001). *p < 0.05, Error bars indicate standard deviation

We examined the expression of miR-663a in normal bronchial cell line HBE135 and 5 NSCLC cell lines: H1299 (adenocarcinoma), H3255 (adenocarcinoma), H460 (large cell carcinoma), H1395 (adenocarcinoma) and H157 (squamous cell carcinoma) using RT-qPCR. As shown in Fig. 2a, compared to the expression in HBE135, downregulation of miR-663a was observed in H157, H1395 and H460 cell lines. H3255 cell line has the same level of miR-663a as HBE135 and H1299 has relative high expression. We explored the potential effects of miR-663a on proliferation, cell cycle progression and invasion in lung cancer cells. According to the expression of miR-663a in NSCLC cell lines, we selected H460 cells for miR-663a mimic transfection and H1299 cells for miR-663a inhibitor transfection. Then we examined the efficiency of miR-663a mimic and inhibitor by real-time PCR at 48 h after transfection. As shown in Fig. 2a, miR-663a mimic significantly upregulated miR-663a in H460 cells and inhibitor downregulated miR-663a expression in H1299 cells. Colony formation assay showed that colony number of H460 cells transfected with miR-663 mimic was significantly decreased (control 596 ± 28 vs mimic 401 ± 18, *p* < 0.05), while colony formation ability of H1299 transfected with miR-663a inhibitor was increased (control 171 ± 16 vs inhibitor 295 ± 18, *p* < 0.05) (Fig. 2b). Cell cycle analysis showed that treatment of miR-663a mimic inhibited G1-S transtion, while miR-663a inhibitor treatment facilitated cell cycle progression (Fig. 2c).

Matrigel invasion assays were also performed, and exogenously increase of miR-663a expression significantly reduced the number of invasive cells by 41.8 % in H460 cells (control 86 ± 6 vs mimic 50 ± 3, *p* < 0.05) (Fig. 2d). The number of invasive cells significantly increased by 48.8 % in H1299 cellstransfected with miR-663a inhibitor compared with the control cells (control 84 ± 5 vs inhibitor 125 ± 9, *p* < 0.05) (Fig. 2d).

**Fig. 3:**
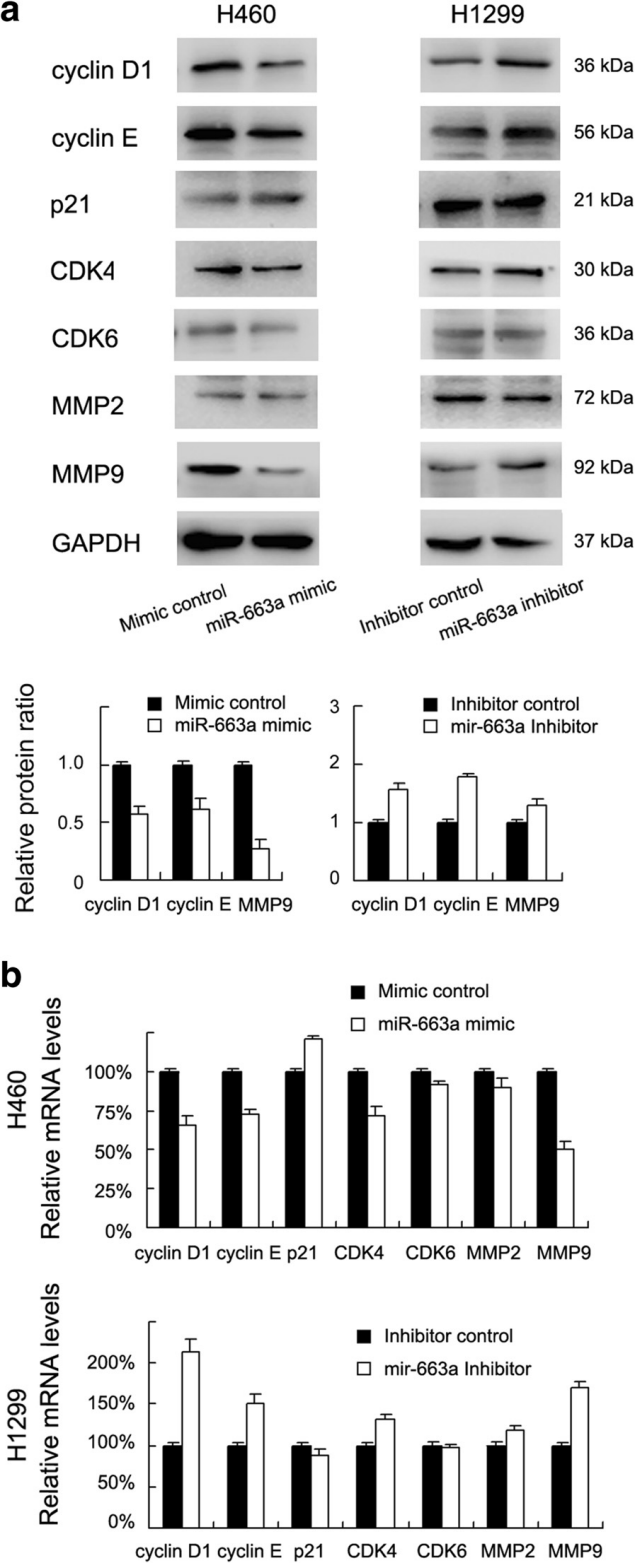
miR-663a regulates mRNA and protein expression of cyclin D1, cyclin E and MMP9. **a** Western blot showed that cyclin D1, cyclin E and MMP9 protein levels of H460 cells treated with miR-663a mimic were lower than those of control. cyclin D1, cyclin E, MMP9 protein levels of H1299 cells treated with miR-663a inhibitor were higher than those of control. Protein quantification of protein was shown for cyclin D1, cyclin E and MMP9 in these two cell lines. **b** Realtime PCR analysis showed that cyclin D1, cyclin E, MMP9 mRNA expression of H460 cells treated with miR-663a mimic were lower than those of cells transfected with control. cyclin D1, cyclin E, MMP9 mRNA expression of H1299 cells treated with miR-663a inhibitor were higher than those of cells transfected with control. Experiments were repeated in triplicate. Error bars indicate standard deviation

In addition, to demonstrate the underlying mechanisms of miR-663a- mediated inhibition of proliferation and invasion, weexamined expression of cell cycle and invasion related proteins, including cyclin D1, cyclin E, p21, CDK4, CDK6, MMP2 and MMP9 by realtime PCR and western blot. The results showed that miR-663a mimic transfection could downregulate cyclin D1, cyclin E and MMP9 expression, while treatment with miR-663a inhibitor upregulated these proteins (Fig. 3a and b). The effect of miR-663a on other proteins was not obvious.

Since the difference in miR-663a levels between H1299 and H460 cells is not large, we also transfected miR-663a inhibitor in H460 cells and miR-663a mimic in H1299 cells to further confirm the results. Consistent with the above findings, we found that colony number of H1299 cells transfected with a mimic was significantly decreased (control 278 ± 20 vs mimic 169 ± 18, *p* < 0.05), while colony formation ability of H460 transfected with miR-663a inhibitor was increased (control 415 ± 32 vs mimic 581 ± 19, *p* < 0.05). Similarly, cell cycle analysis showed that treatment of miR-663a mimic in H1299 cells inhibited G1-S transtion, while miR-663a inhibitor treatment in H460 cells facilitated cell cycle progression. In addition, exogenously upregulation of miR-663a in H1299 cells reduced the number of invasive cells significantly (control 72 ± 3 vs mimic 46 ± 4, *p* < 0.05). The number of invasive cells in H460 cells increased significantly when transfected with miR-663a inhibitor (control 45 ± 3 vs mimic 61 ± 4, *p* < 0.05). Finally, we assessed the roles of miR-663a in the regulation of cell-cycle and cell-invasion related molecules. As expected, miR-663a inhibitor increased both protein and mRNA expression of cyclin D1, cyclin E, MMP9 and JunD in H460 cells, meanwhile miR-663a mimic showed opposite effects in H1299 cells (Additional file 1: Figure S1).

### miR-663a inhibits AP-1 transcription activity by targeting JunD

**Fig. 4:**
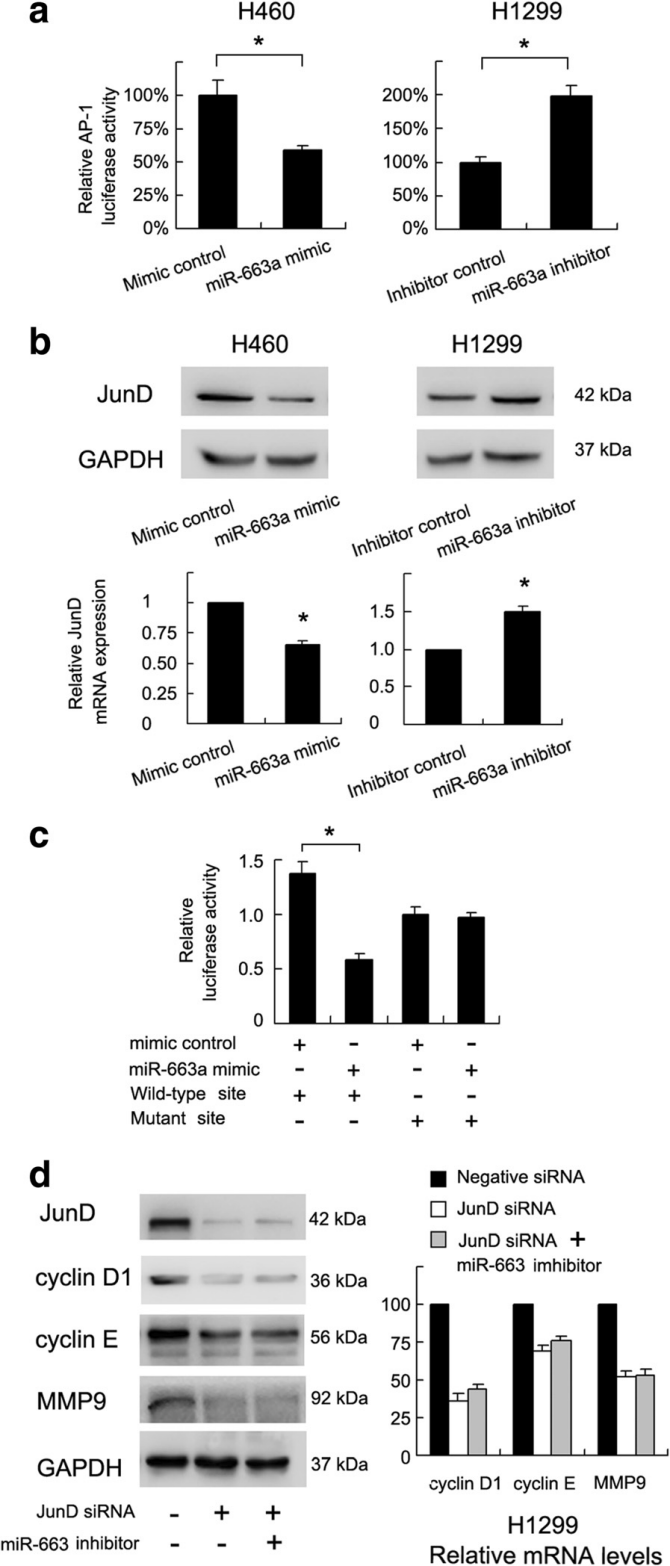
miR-663a targeted AP-1 component JunD in NSCLC cells. **a** In H460 cells transfected with miR-663a mimic, the AP-1 activity was lower than that in H460 cells transfected with control. The AP-1 activity in H1299 transfected with miR-663a inhibitor was higher than that transfected with control. **b** JunD protein and mRNA expression in H460 cells treated with miR-663a mimic was lower than that in H460 cells transfected with control. JunD protein and mRNA expression in H1299 treated with miR-663a inhibitor was higher than that in H1299 cells transfected with control. **c** Wild-type and mutant miR-663a binding sites in the 3′-UTR of JunD were assessed using fluorescent reporter assays. The fluorescence activity of miR-663a mimic/mimic control was calculated. In H460 cells transfected with reporter vector containing wild-type binding site, the luciferase activity in cells with miR-663a mimic was lower than that in control cells. **d** JunD siRNA treatment in H1299 cells downregulated protein and mRNA expression of cyclin D1, cyclin E and MMP9. In JunD depleted cells, difference of cyclin D1, cyclin E, MMP9 mRNA in cells treated with control and miR-663a inhibitor was not significant. Experiments were repeated in triplicate. *p < 0.05, Error bars indicate standard deviation

Since miR-663a could downregulate cyclin D1, cyclin E and MMP9 expression, we examined several signaling pathways which could regulate these genes simultaneously. Using luciferase reporter plasmid, we found that miR-663a mimic transfection inhibited AP-1 transcription activity and miR-663a inhibitor increased AP-1 activity (Fig. 4a).

To determine potential target genes of miR-663a in NSCLC, the TargetScan Human database 6.2 was used. Among the genes in the target list, we focused on JunD which is an AP-1 component and a key mediator of tumor cell proliferation, metastasis and chemoresistance in a variety of tumors (Additional file 2: Figure S2). To evaluate whether JunD is regulated by miR-663a in vitro, we transfected H460 cells with miR-663a mimic and detected the mRNA and protein levels of JunD 48 h after transfection.. As shown in Fig. 4, ectopic expression of miR-663a significantly decreased both mRNA and protein expression of JunD compared to controls., indicating miR-663a as a potent regulator of JunD.

To further determine if JunD was a direct target of miR-663a, fluorescent reporter assays were performed. According to a previous study [15], the 3′-UTR of JunD with wild-type (CCCCGCC) and mutant (CGGGCGC) binding sites for miR-663a was cloned into pmiR-RB-REPORT vector. The ratio of fluorescence intensity for wild-type and mutant binding sites was calculated. As shown in Fig. 4c, miR-663a mimic reduced the fluorescence intensity in H460 cells transfected with a vector containing wild-type JunD 3′-UTR compared with controls, while no significant change was observed in cells transfected with vector containing mutant binding site. These results indicated that miR-663a binds to JunD 3′-UTR region directly and downregulates JunD mRNA expression. To validate this, we analyzed the relationship between miR-663a and JunD mRNA expression in NSCLC tissues using linear regression. We found that miR-663a negatively correlated with JunD mRNA expression (Fig. 1f, *p* = 0.005).

To confirm the role of JunD-AP1 axis in miR-663a induced inhibition of cyclin proteins and MMP9. We knocked down JunD using siRNA in H1299 cell line. Western blot and realtime PCR analysis showed that JunD depletion downregulated mRNA and protein expression of cyclin D1, cyclin E and MMP9. In addition, in JunD siRNA treated H1299 cells, miR-663a inhibitor failed to upregulate cyclin D1, cyclin E and MMP9. These results indicated the pivotal role of JunD in miR-663a mediated inhibition of cell growth and invasion.

## Discussion

Accumulated evidence in the literature implicates an important role of miR-663a in cancer development [14–17]. It was reported that expression of miR-663a was significantly downregulated in pediatric AML cells, which may caused by hypermethylation of the miR-663a promoter [17]. Downregulation of miR-663a was also observed in gastric cancers and introduction of miR-663a into the human gastric cancer cells suppressed proliferation and mitotic catastrophe [14]. In addition, the tumor suppressive role of miR-663a was demonstrated in colorectal cancer cells [16], prostate cancer and especially in glioblastoma, in which miR-663a targeted PIK3CD and served as a prognostic biomarker [20]. However, the involvement of miR-663a in tumor progression remains controversial and some reports implied miR-663a as a oncogene in breast and nasopharyngeal carcinoma [18, 19]. So far, little is known about the expression pattern of miR-663a in NSCLC and its effects on tumor invasion and cell cycle progression. In the present study, we found that miR-663a was significantly downregulated in NSCLC tissues and cell lines by RT-qPCR analysis, which is consistent with previous reports [14, 17, 18, 20]. In addition, statistical analysis showed that miR-663a downregulation correlated with lymph node metastasis. The level of miR-663a was also lower in several lung cancer cell lines compared with normal bronchial cell line HBE135. These results suggested that downregulation of miR-663a might be a useful predictor of NSCLC.

The precise molecular mechanisms for the altered expression of miR-663a in lung cancers remain unknown. It was reported hypermethylation of miR-663apromoter, which could be affected by 5-Aza demethylation,was observed in acute myeloid leukemia [17]. Thus, aberrant epigenetic events may contribute to miR-663a dysregulation in lung cancer and this needs further studies.

The biological functions of miR-663a in lung cancer cells and the possible mechanisms were investigated. Cell cycle analysis and colony formation assays showed that miR-663a inhibited cell growth and induced cell cycle arrest, and importantly,a series of cell cycle regulators, including cyclin D1 and cyclin E, were affected by miR-663a. The correlation of low miR-663a expressionwith lymph node metastasis indicated that, as a potential tumor suppressor, miR-663a might not only regulate cell cycle but also invasion and tumor metastasis by regulating multiple oncogenes. As expected, transwell assays revealed that miR-663a suppressed cell invasion, and western blot results showed that MMP9, a criticalinvasion regulator, was downregulated after induction of miR-663a mimic and upregulated with miR-663a inhibitor treatment. These results suggested that miR-663a suppresses cell growth and invasion through inhibition of Cyclins and MMP9 expression.

AP-1 signaling was reported to regulate cyclin D1, cyclin E and MMP9 in various cancers including NSCLC [7, 21]. Using luciferase reporter plasmid, we found that miR-663a inhibited AP-1 transcription activity. So among the potential miR-663a targets list predicted by TargetScan Human database, we sparked a strong interested in JunD which is a functional component of the AP1 transcription factor complex and also a proved target of miR-663a in human THP-1 monocytic cell [15]. In vitro overexpression of miR-663a significantly decreased JunD mRNA and protein expression compared to controls, whereas inhibition of miR-663a led to increased JunD mRNA and protein. Correspondingly, we also found a negative correlation between miR-663a and JunD mRNA in NSCLC tissues. Furthermore, fluorescent reporter assays demonstrated that miR-663a directly bound to the JunD 3′-UTR region, which was in accordance with previous report [15]. As an AP-1 transcription factor, JunD was reported to accelerate growth, inhibit apoptosis and enhance cancer cell invasion [22–26]. Using siRNA knockdown, we confirmed that JunD downregulation decreased expression of cyclin D1, cyclin E and MMP9 in lung cancer cells. JunD siRNA also abrogated the effect of miR-663a inhibitor. Thus our results indicated that miR-663a inhibited cell cycle and invasion, at least partly, through regulation of JunD expression.

## Conclusions

In conclusion, miR-663a, a downregulated miRNA in NSCLC, was associated with lymph node metastasis. In addition, miR-663a regulated cell cycle and invasion by targeting AP-1 component JunD, which provides new insights into the molecular mechanisms of lung cancer progression.

### Ethics approval and consent to participate

This study was conducted with the approval of the Ethics Committee at Shengjing Hospital of China Medical University. Written informed consent was obtained from all patients. Research carried out is in compliance with the Helsinki Declaration.

### Consent for publication

Not applicable

### Availability of data and materials

The dataset supporting the conclusions of this article is available in the following repository: http://pan.baidu.com/s/1i4YrsrF

## Additional files

Additional file 1: Figure S1. A. Colony number of H460 cells transfected with a inhibitor was higher than cells transfected with control (control 415 ± 32 vs mimic 581 ± 17, *p* < 0.001). Colony number of H1299 transfected with miR-663a mimic was lower than cells transfected with control (control 278 ± 20 vs inhibitor 169 ± 18, *p* < 0.001). B. Matrigel invasion assay showed that invading number of H460 transfected with miR-663a inhibitor was higher that cells transfected with control (control 45 ± 3 vs mimic 61 ± 4, *p* = 0.007). Invading number of H1299 transfected with miR-663a mimic was lower that cells transfected with control (control 72 ± 3 vs inhibitor 46 ± 4, *p* < 0.001). C. Western blot showed that cyclin D1, cyclin E, MMP9, JunD protein levels of H460 cells treated with miR-663a inhibitor were higher than those of control. cyclin D1, cyclin E, MMP9, JunD protein levels of H1299 cells treated with miR-663a mimic were lower than those of control. D. Realtime RT-PCR showed that cyclin D1, cyclin E, MMP9, JunD mRNA levels of H460 cells treated with miR-663a inhibitor were higher than those of control. cyclin D1, cyclin E, MMP9, JunD mRNA levels of H1299 cells treated with miR-663a mimic were lower than those of control. Experiments were repeated in triplicate. Error bars indicate standard deviation. (TIFF 1946 kb) Additional file 2: Figure S2. The JunD information from the TargetScan Human database. The information is from the TargetScan Human database. (TIFF 42 kb)

## Abbreviations

3′-UTR: three prime untranslated region
miRNA: microRNA
NSCLC: non-small cell lung
RT-qPCR: reverse transcription quantitative polymerase chain reaction
