# Supplementary figures and images for: MicroRNA-663a is downregulated in non-small cell lung cancer and inhibits proliferation and invasion by targeting JunD
Source: BMC Cancer. 2016 May 16;16:315. doi: 10.1186/s12885-016-2350-x (PMC4869303; doi:10.1186/s12885-016-2350-x)

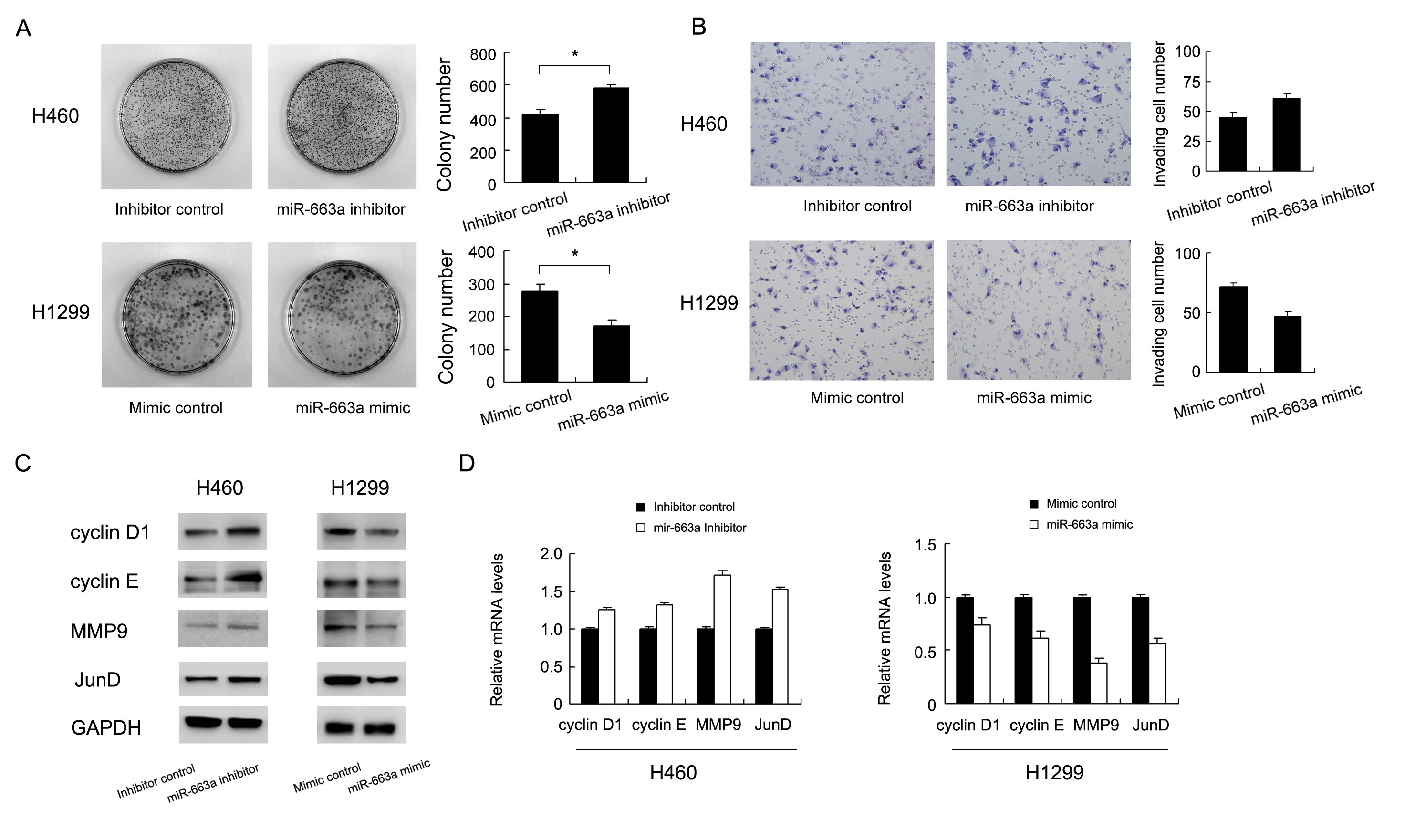

Supplement: Additional file 1: Figure S1. — A. Colony number of H460 cells transfected with a inhibitor was higher than cells transfected with control (control 415 ± 32 vs mimic 581 ± 17, p < 0.001). Colony number of H1299 transfected with miR-663a mimic was lower than cells transfected with control (control 278 ± 20 vs inhibitor 169 ± 18, p < 0.001). B. Matrigel invasion assay showed that invading number of H460 transfected with miR-663a inhibitor was higher that cells transfected with control (control 45 ± 3 vs mimic 61 ± 4, p = 0.007). Invading number of H1299 transfected with miR-663a mimic was lower that cells transfected with control (control 72 ± 3 vs inhibitor 46 ± 4, p < 0.001). C. Western blot showed that cyclin D1, cyclin E, MMP9, JunD protein levels of H460 cells treated with miR-663a inhibitor were higher than those of control. cyclin D1, cyclin E, MMP9, JunD protein levels of H1299 cells treated with miR-663a mimic were lower than those of control. D. Realtime RT-PCR showed that cyclin D1, cyclin E, MMP9, JunD mRNA levels of H460 cells treated with miR-663a inhibitor were higher than those of control. cyclin D1, cyclin E, MMP9, JunD mRNA levels of H1299 cells treated with miR-663a mimic were lower than those of control. Experiments were repeated in triplicate. Error bars indicate standard deviation. (TIFF 1946 kb) [file 12885_2016_2350_MOESM1_ESM.tiff]

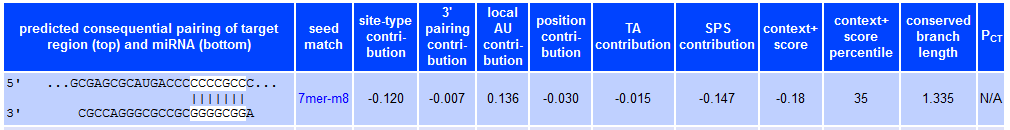

Supplement: Additional file 2: Figure S2. — The JunD information from the TargetScan Human database. The information is from the TargetScan Human database. (TIFF 42 kb) [file 12885_2016_2350_MOESM2_ESM.tiff]
